# Chloroquine and bafilomycin A mimic lysosomal storage disorders and impair mTORC1 signalling

**DOI:** 10.1042/BSR20200905

**Published:** 2020-04-28

**Authors:** Anthony O. Fedele, Christopher G. Proud

**Affiliations:** Hopwood Centre for Neurobiology, Lifelong Health Theme, South Australian Health and Medical Research Institute (SAHMRI), PO Box 11060, Adelaide 5001, South Australia, Australia

**Keywords:** autophagy, bafilomycin A, Chloroquine, lysosomal storage disorders, lysosome, mammalian target of rapamycin complex 1 (mTORC1)

## Abstract

Autophagy is dependent upon lysosomes, which fuse with the autophagosome to complete the autophagic process and whose acidic interior permits the activity of their intraluminal degradative enzymes. Chloroquine (CQ) and bafilomycin A1 (BafA) each cause alkalinisation of the lumen and thus impair lysosomal function, although by distinct mechanisms. CQ diffuses into lysosomes and undergoes protonation, while BafA inhibits the ability of the vacuolar type H^+^-ATPase (v-ATPase) to transfer protons into the lysosome. In the present study, we examine the impact of CQ and BafA on the activity of mammalian target of rapamycin complex 1 (mTORC1), inhibition of which is an early step in promoting autophagy. We find each compound inhibits mTORC1 signalling, without affecting levels of protein components of the mTORC1 signalling pathway. Furthermore, these effects are not related to these agents’ capacity to inhibit autophagy or the reduction in amino acid supply from lysosomal proteolysis. Instead, our data indicate that the reduction in mTORC1 signalling appears to be due to the accumulation of lysosomal storage material. However, there are differences in responses to these agents, for instance, in their abilities to up-regulate direct targets of transcription factor EB (TFEB), a substrate of mTORC1 that drives transcription of many lysosomal and autophagy-related genes. Nonetheless, our data imply that widely used agents that alkalinise intralysosomal pH are mimetics of acute lysosomal storage disorders (LSDs) and emphasise the importance of considering the result of CQ and BafA on mTORC1 signalling when interpreting the effects of these agents on cellular physiology.

## Introduction

The mammalian target of rapamycin complex 1 (mTORC1) is a heteromeric protein kinase which phosphorylates a diverse array of substrates. It thereby activates multiple anabolic cellular processes, including protein synthesis, lipogenesis, and ribosome biogenesis, while also inhibiting catabolic processes, in particular, autophagy [1]. mTORC1 phosphorylates the protein kinase Unc-51 like autophagy activating kinase 1 (ULK1), thereby inhibiting its ability to stimulate autophagy [2]. In addition, the transcription factor EB (TFEB), which promotes transcription of numerous genes encoding proteins involved in autophagy, is inhibited as a result of its phosphorylation by mTORC1 [3] and thus promotes lysosomal biogenesis [4,5]. Thus, mTORC1 suppresses autophagy in two distinct and complementary ways: one acting acutely (via ULK1) and the other over a longer term (TFEB).

Autophagy requires the function of lysosomes whose acidic interior permits the activity of their intraluminal degradative enzymes, such as acidophilic proteases. Furthermore, mTORC1 is activated on the cytoplasmic surface of lysosomes by two different types of GTPases; Rheb mediates the activation of mTORC1 in response to hormones and growth factors that act via cell surface receptors, while the Rag GTPases relay information about the availability of amino acids, which positively regulate mTORC1 signalling [6]. The Rag GTPases are associated with Ragulator complex, which is tethered to the lysosome through the vacuolar type H^+^-ATPase (v-ATPase) [7]. The significance of lysosomes as the platform on which mTORC1 is regulated may, at least in part, reflect the fact that proteolysis within the lysosome could provide amino acids to help sustain mTORC1 activity [8–10]. Given that mTORC1 controls autophagy, another role for the lysosomal association of mTORC1 may be to allow mTORC1 to sense autophagic/lysosomal function and be regulated by it in times of lysosomal dysfunction, such as during reduced clearance of substrates [11].

Chloroquine (CQ) alkalinises the lumen of the lysosome and thus inhibits its pH-sensitive functions, although its mode of action is yet to be conclusively elucidated. The most likely explanation is that CQ enters the lysosome by diffusion, but upon protonation to CQ^2+^, cannot diffuse out of the intralysosomal environment [12]. By mopping-up protons, this compound increases intralysosomal pH, and inhibits both intralysosomal proteolysis and fusion of the autophagosome with the lysosome (and ultimately autophagy) [12,13]. Indeed, the utility of CQ as a pharmaceutical agent to treat malaria is based on its acidotropic nature, and its characteristic of entering, but, when protonated, being unable to leave the digestive vacuole of this parasite when they are inside red blood cells. In this case, however, CQ forms a complex with haem, which in turn disrupts membrane function to the point of high toxicity to the malarial protozoan [14,15]. Recent interest has focused on the utility of CQ derivatives such as hydroxychloroquine in treating infections of the novel coronavirus termed as COVID-19 which have reached pandemic status [16]. Bafilomycin A1 (BafA) de-acidifies the lysosome in an indirect way by inhibiting the v-ATPase [17,18], which normally transfers protons into the lysosome to maintain its low pH.

Given their very widespread use in diverse studies over many years, we felt it important to examine the possible impact of CQ and BafA on mTORC1 signalling. We find that each causes a reduction in mTORC1 activity. Furthermore, our data imply that agents that alkalinise intralysosomal pH should be considered not only inhibitors of autophagy, but rather as mimetics of acute lysosomal storage disorders (LSDs), in which reduced mTORC1 signalling has previously been implicated. Therefore, the impact of BafA and CQ on mTORC1 signalling should be taken into account when interpreting the effects of these widely used agents on cellular or organismal physiology. Furthermore, we also report significant differences in the responses to these two agents on the 5′-AMP activated protein kinase (AMPK), another key regulator of cellular functions.

## Materials and methods

### Chemicals

AZD8055 (Selleck Chem), bafilomycin A (Sigma–Aldrich), and rapamycin (Sigma–Aldrich) were all dissolved in DMSO, while CQ diphosphate salt (Sigma–Aldrich), leupeptin hydrochloride (Sigma–Aldrich), and sucrose (Sigma–Aldrich) were prepared in Milli-Q water. A total of 50× MEM essential amino acids and 100× MEM non-essential amino acids were supplied by Thermo Fisher.

### Cell culture

Human lung carcinoma A549 and cervical cancer HeLa cells were grown in Dulbecco’s modified Eagle’s medium (DMEM, Sigma), supplemented with 10% (v/v) foetal calf serum (Life Technologies), 100 U/ml penicillin/streptomycin (Sigma), and incubated at 37°C and 5% (^v^/_v_) CO_2_.

### Transient transfection

siRNA to *BECN1* (Santa Cruz) was introduced into cells using Lipofectamine 3000 (Life Technologies) according to the manufacturer’s instructions.

### Immunoblotting

Cells were plated on 9-cm^2^ wells. Upon completion of described treatments, the medium was removed, and cells were washed with 2 ml of PBS, after which 250 µl of RIPA buffer supplemented with 2.5 mM of Na_2_H_2_P_2_O_7_ (Sigma–Aldrich), 1 mM β-glycerophosphate (Sigma–Aldrich), 1 mM Na_3_VO_4_ (Sigma–Aldrich), and 1× Protease Inhibitor Cocktail (Roche) was added. Cells were then rocked for 30 min at 4°C. Cells were harvested into microfuge tubes and centrifuged at 13000×***g*** for 30 min at 4°C. The supernatant was maintained and transferred to a new microfuge tube. The protein concentration of each sample was determined via Lowry [19]. Identical amounts of quantified proteins of each were then electrophoresed through a 4–12% (w/v) acrylamide gradient SDS/PAGE gel and transferred to a PVDF membrane.

Membranes were incubated for 1 h at room temperature in PBS containing 0.1% Tween 20 and 2% bovine serum albumin (BSA) and then rocked overnight at 4°C in the same solution supplemented with the primary antibody at a 1/1000 dilution (unless otherwise stated). The following day, the membrane was washed three times for 5–10 min at room temperature in PBS containing 0.1% Tween 20, then incubated at room temperature for 1 h in PBS containing 0.1% Tween 20 and 2% BSA plus 1/3000 horseradish peroxidase-linked sheep anti-mouse (GE Healthcare), goat anti-rabbit (Millipore), or 1/5000 mouse anti-human β-actin (Sigma) IgG. The membrane was then washed three times for 5–10 min at room temperature in PBS with 0.1% Tween 20, drained, treated with the Supersignal West Pico Plus Chemiluminescent Substrate (Thermo Fisher), developed on an LAS-4000 apparatus (Fujifilm), and analysed using Multigauge 1D software (Fujifilm). In some cases, further densitometric quantification was implemented with ImageJ (NIH). The following primary antibodies were supplied by Cell Signaling Technology: rabbit polyclonal anti-human ribosomal protein S6 (rpS6) P-S240/244, rabbit polyclonal anti-human p70S6K1 P-T389, rabbit polyclonal anti-human p62, rabbit polyclonal anti-human acetyl-CoA carboxylase (ACC), and rabbit polyclonal anti-rat ACC P-S79. Mouse monoclonal anti-human rpS6, rabbit polyclonal anti-rat p70S6K1, rabbit polyclonal anti-human TSC1 (used at 1/200 dilution) and rabbit polyclonal anti-human TSC2 (used at 1/200 dilution) were purchased from Santa Cruz. Rabbit polyclonal anti-human microtubule-associated proteins 1A/1B light chain 3B (LC3) and rabbit polyclonal anti-human beclin-1 were provided by Novus Biologicals. Rabbit polyclonal anti-human RAPTOR was custom made by MRC PPU Reagents (University of Dundee) while monoclonal anti-human lysosomal-associated membrane protein 1 (LAMP-1) was raised in-house.

### Immunofluorescence microscopy

Cells were plated on four-well Lab-Tek II chamber slides (Thermo Scientific). Upon completion of described treatments, cells were rinsed three times in PBS, then fixed for 15 min in 4% PFA. Fixed cells were washed three times for 5 min at room temperature in PBS and permeabilised for 5 min in PBS containing 0.05% Triton X-100. Cells were then washed three times for 5 min at room temperature in PBS and blocked for 30 min at room temperature in 10% normal donkey serum (Jackson ImmunoResearch). This was replaced with 2% normal donkey serum containing primary antibodies at appropriate concentrations and incubated at room temperature for 1 h. Cells were washed three times for 5 min in PBS and secondary antibodies in 2% normal donkey serum were then applied and incubated at room temperature for 1 h. Cells were washed three times for 5 min in PBS and slides mounted in VectaShield with DAPI (Vector Laboratories). The following antibodies were used at 1/200 dilution: sheep anti-human LAMP-1 (raised in-house), rabbit anti-human mTOR (Cell Signaling Technology), donkey anti-rabbit Alexa Fluor 488 (Jackson ImmunoResearch), and donkey anti-sheep Alexa Fluor 594 (Jackson ImmunoResearch). Slides were examined and imaged on a Leica TCS SP8X confocal microscope. Images were analysed using the Fiji package [20]. Mander’s coefficients were determined using the JACoP function [21] of ImageJ (NIH).

### Magic red and lysotracker microscopy

Cells were plated on four-well Lab-Tek II chamber slides (Thermo Scientific). One hour prior to the completion of described treatments, growth medium was also supplemented with Magic Red Cathepsin L Kit (Bio-Rad) or LysoTracker Red DND-99 (Life Technologies) according to the manufacturers’ instructions. Cells were rinsed three times in PBS, then fixed for 15 min in 4% PFA. Fixed cells were washed three times for 5 min at room temperature in PBS and slides mounted in VectaShield with DAPI (Vector Laboratories). Slides were examined and imaged on a Leica TCS SP8X confocal microscope. To permit a comparison, the exposure time for the detection of Magic Red or LysoTracker Red DND-99 was maintained at a constant. Images were analysed using the Fiji package [20].

### Quantitative real-time PCR

Cells were plated on to 9-cm^2^ wells. Upon completion of the described treatments, medium was removed, and cells were washed with 2 ml of PBS, after which 500 µl of TRIzol reagent (Life Technologies) was added. Total RNA was subsequently prepared according to the manufacturer’s instructions, 500 ng of which provided a template for the preparation of complementary DNA (cDNA) using the SuperScript III First Strand Synthesis System (Life Technologies). The cDNA was then analysed via quantitative real-time PCR (qPCR) employing primers (Table 1) designed to amplify fragments of the coding sequences of the lysosomal membrane protein LAMP-1 (Entrez nucleotide accession number NM_005561), the autophagy-related proteins BECN1 (Entrez nucleotide accession number NM_003766.4), and p62/SQSTM1 (Entrez nucleotide accession number NM_003900.4), as well as the internal control β-actin. This entailed preparing a mixture containing the equivalent of 25 ng of the original template RNA in 1× Fast SYBR Green Mix (Applied Biosystems) and 200 nM of the relevant reverse and forward primer oligonucleotides (Table 1). qPCRs proceeded as follows on an ABI Step One Plus qPCR instrument (Applied Biosystems): 95°C for 20 s, 40× (95°C for 3 s, 60°C for 30 s). The comparative threshold cycle protocol was employed to determine amounts of the target mRNA.

**Table 1:** Oligonucleotides employed in the present study

Name	Sequence
actin_320_for	5′-CTGGCACCACACCTTCTAC-3′
actin_557_rev	5′-GGGCACAGTGTGGGTGAC-3′
BECN1_106_for	5′-CGTGTCACCATCCAGGAAC-3′
BECN1_286_rev	5′-TGTTGGCACTTTCTGTGGAC-3′
LAMP1_352_for	5′-ATTGTGCGTCAGCAGCAATG-3′
LAMP1_505_rev	5′-AGCACCACTGTGGCATCTG-3′
p62_684_for	5′-ATCGGAGGATCCGAGTGTG-3′
p62_846_rev	5′-GCTCTTCTCCTCTGTGCTG-3′

### Statistics

For data represented graphically, error bars represent ± standard error of the mean of the indicated number of independent experiments performed. Statistical significance was determined using unpaired Student’s *t* test. *P≤0.05, **P≤ 0.01, ***P≤0.001, ****P≤0.0001.

## Results

### Agents that cause lysosomal alkalinisation impair mTORC1 signalling

**Figure 1:**
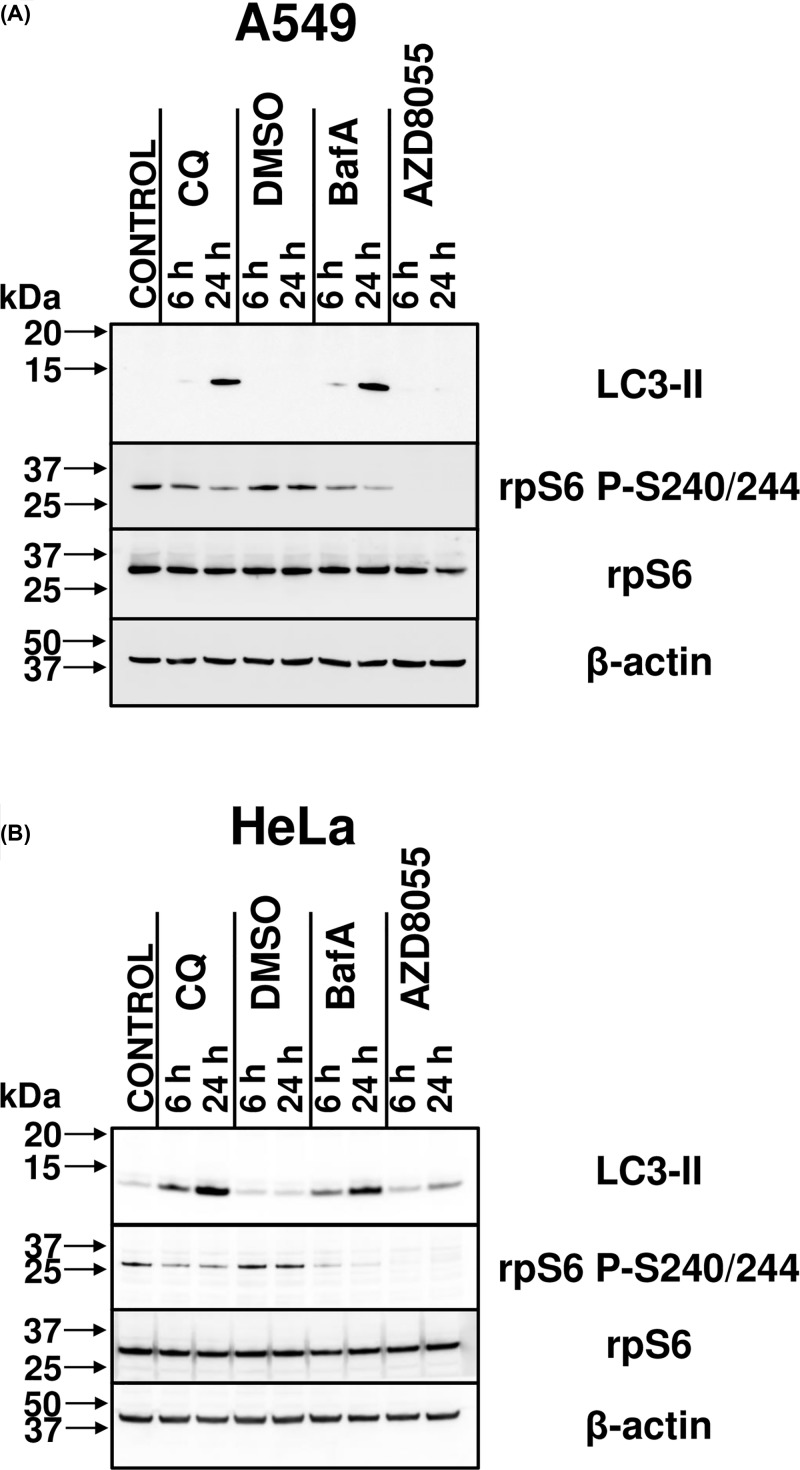
BafA and extended CQ treatment reduces ribosomal protein S6 phosphorylation in A549 and HeLa cells (**A**) Cell extracts from the human lung carcinoma A549 cells which had been treated with 1:1000 DMSO (vehicle), 1 µM AZD8055, 50 µM CQ, or 200 nM BafA, or left untreated (CONTROL) for 6 or 24 h were analysed by immunoblotting with antibodies to LC3-II (a component of the autophagosome), total and phospho-rpS6 (an indirect effector of mTORC1 kinase activity, via p70S6K) and β-actin. (**B**) As for (A), but using human cervical cancer HeLa cells. Results are representative of three independent experiments. Replicate experiments are shown in Supplementary Figure S1.

BafA interferes with lysosomal function by inhibiting the v-ATPase, thereby promoting alkalinisation of the lysosomal lumen [15,16]. We thus first examined whether treating cells with BafA affected mTORC1 signalling by applying the commonly used procedure of assessing the phosphorylation of its downstream effectors in whole cell extracts via immunoblotting [7,8], which is more informative regarding intracellular mTORC1 activity than attempting to measure the activity of mTORC1 after isolation from cells which does not yield reliable results. To this end, human lung carcinoma A549 and cervical cancer HeLa cells were treated with 1 µM AZD8055 (an inhibitor of mTOR kinase activity, and thus a control that impairs mTOR signalling) or 200 nM BafA (a commonly used concentration [22] and within the range recommended by manufacturers) for 6 or 24 h. Cells were lysed and samples immunoblotted for LC3-II, a protein that is incorporated into the membrane of autophagosomes during their formation upon processing of LC3-I [23]. It thus serves as a positive control for the efficacy of BafA, as its levels are a marker for the presence of autophagosomes, which accumulate during lysosomal alkalinisation because of the inhibition of fusion of the autophagosome with the lysosome. As expected, BafA treatment caused LC3-II levels to become detectable after 6 h of treatment and increase markedly after 24 h of treatment (Figure 1).

The same samples were also probed for rpS6. Its phosphorylation serves as a robust readout of mTORC1 activity, since rpS6 is phosphorylated at Serine 240 and 244 specifically by p70S6K, which is itself activated by direct phosphorylation of Thr^389^ by mTORC1 [24–26]. BafA caused a reduction in the levels of rpS6 P-S240/244 (relative to total rpS6), the effect increasing at the later time point. This indicates that BafA induces an impairment of mTORC1 signalling.

Since the v-ATPase interacts with Ragulator, which controls mTORC1, it was important to distinguish whether the effects of BafA were due to lysosomal alkalinisation or only to an effect on the v-ATPase/Ragulator axis. Cells were thus also treated with the regularly used dose of 50 µM CQ [27], which directly increases lysosomal pH. In addition to the expected increase in LC3-II levels, the phosphorylation of rpS6 was reduced in a manner similar to that seen with BafA (Figure 1).

**Figure 2:**
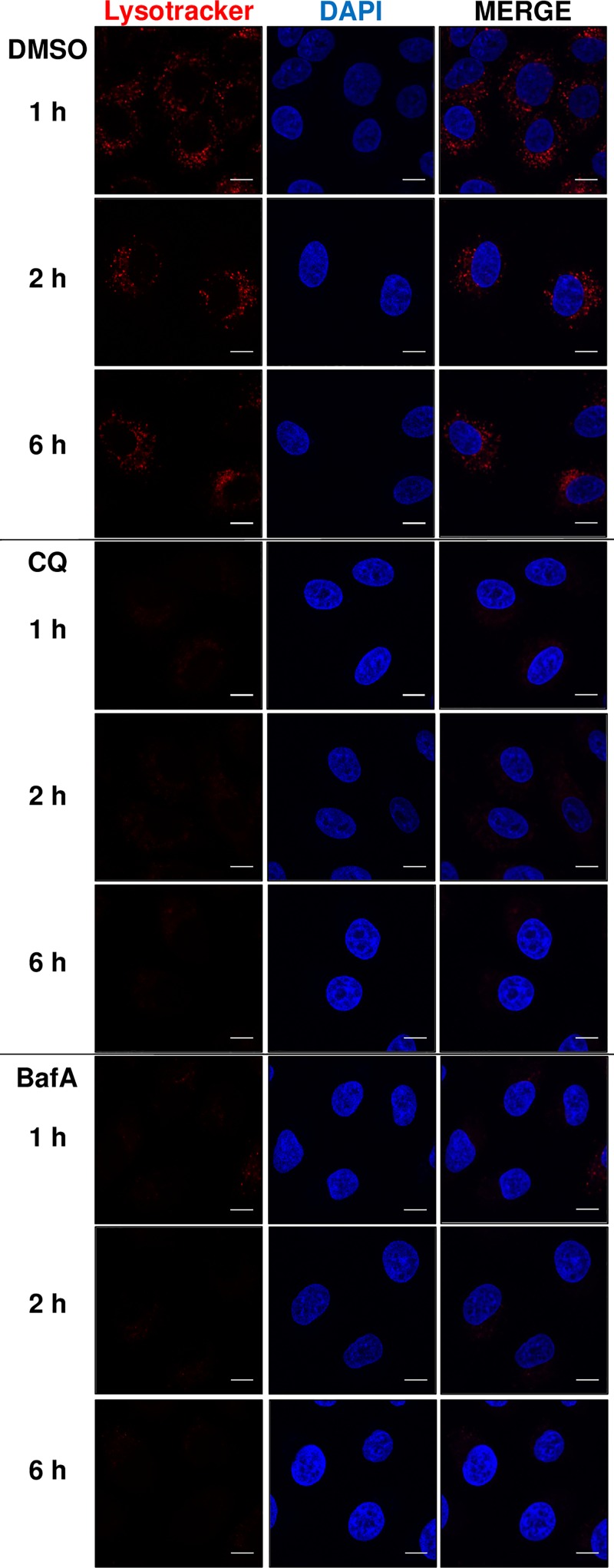
CQ and BafA treatment acutely increase intralysosomal pH A549 cells were treated with 1:1000 DMSO (vehicle), 50 µM CQ, or 200 nM BafA, for 1, 2, or 6 h. Culture medium was additionally supplemented with LysoTracker Red DND-99 for 1 h prior to fixing. All samples were then analysed via confocal microscopy. Each panel represents a set of cells labelled with DAPI (blue) and LysoTracker Red DND-99 (red). The scale bar represents 10 µm. Results are representative of three independent experiments.

There is some controversy with respect to the capacity of CQ to alkalinise lysosomes. For instance, it has been observed previously that treatment of HeLa cells for 5 h with CQ resulted in no decrease in acidity of the lysosome, as determined by the use of LysoTracker Red DND-99, a fluorescent probe that can permeate cell membranes and label acidic organelles (specifically, the lysosome) [13]. To confirm and assess the ability of CQ or BafA to alkalinise the lysosome, A549 cells were treated for 1, 2, and 6 h with either of these chemicals. Prior to fixing the cells, medium was supplemented with LysoTracker Red DND-99. Detection of LysoTracker-stained lysosomes was ablated (Figure 2) as early as the 1 h time-point with either CQ or BafA, indicating that alkalinisation precedes impairment of mTORC1 signalling by several hours. Indeed, there was no reduction in rpS6 phosphorylation at the 1 and 2 h time points of CQ and BafA treatment (Supplementary Figure S2). This delay in itself implies that reduced mTORC1 activity is not a direct consequence of lysosomal alkalinisation itself and/or (with respect to BafA) inhibition of the v-ATPase, as those effects of the compounds are much more rapid.

**Figure 3:**
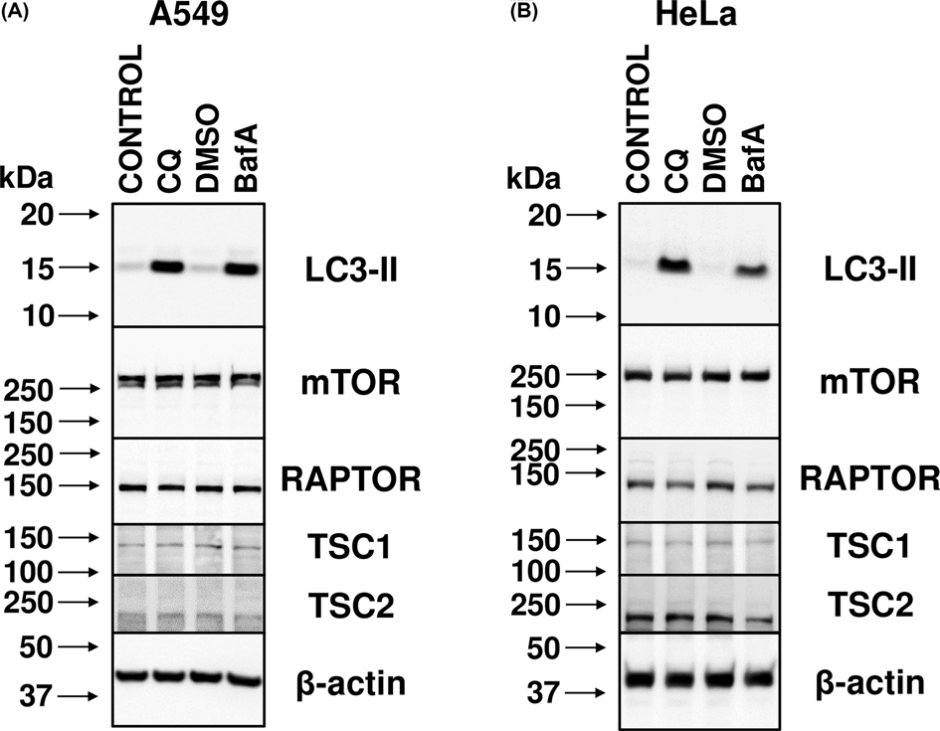
CQ and BafA do not change the levels of proteins involved in mTORC1 signalling (**A**) A549 cells treated with 1:1000 DMSO (vehicle), 50 µM CQ, or 200 nM BafA, or left untreated (CONTROL) for 24 h were analysed by immunoblotting with antibodies to the indicated antibodies. (**B**) As for (A), but using HeLa cells. Results are representative of three independent experiments.

Finally, although it was not anticipated that this would be the case, to confirm whether treatment with CQ or BafA affects the levels of expression of components of mTORC1 or proteins that regulate it, extracts from A549 and HeLa cells treated with CQ or BafA for 24 h were immunoblotted with antibodies to mTOR, RAPTOR (the defining component of mTORC1), tuberous sclerosis (TSC) 1 and TSC2 (negative regulators of mTORC1). Supplementation of the medium with CQ and BafA expectedly increased LC3-II levels, but there were no significant changes in the levels of the other proteins studied (Figure 3). It is thus unlikely that lysosomal alkalinisation impairs mTORC1 signalling by affecting the expression of mTORC1 components mTOR and RAPTOR, or its upstream regulators TSC1/2, but rather impacts the regulation of mTORC1.

The above data show that raising the intralysosomal pH occurs within a comparable timeframe in response to CQ or BafA, and impacts negatively on mTORC1 signalling. Given that these phenomena also result from CQ treatment, this also means that they occur independently of interference with the v-ATPase. However, importantly, the decline in mTORC1 activity appears not to be directly due to either inhibition of the v-ATPase or the change in pH, as these effects are faster than the reduction in mTORC1 signalling. It is thus likely an indirect consequence of altered luminal pH.

### CQ and BafA treatment results in reduced localisation of mTOR with the lysosome

As mTORC1 is activated on the cytoplasmic surface of lysosomes [8], we asked whether CQ or BafA altered its intracellular distribution. We thus evaluated the co-localisation of endogenous mTOR with a marker for lysosomes, lysosomal-associated membrane protein (LAMP)-1.

**Figure 4:**
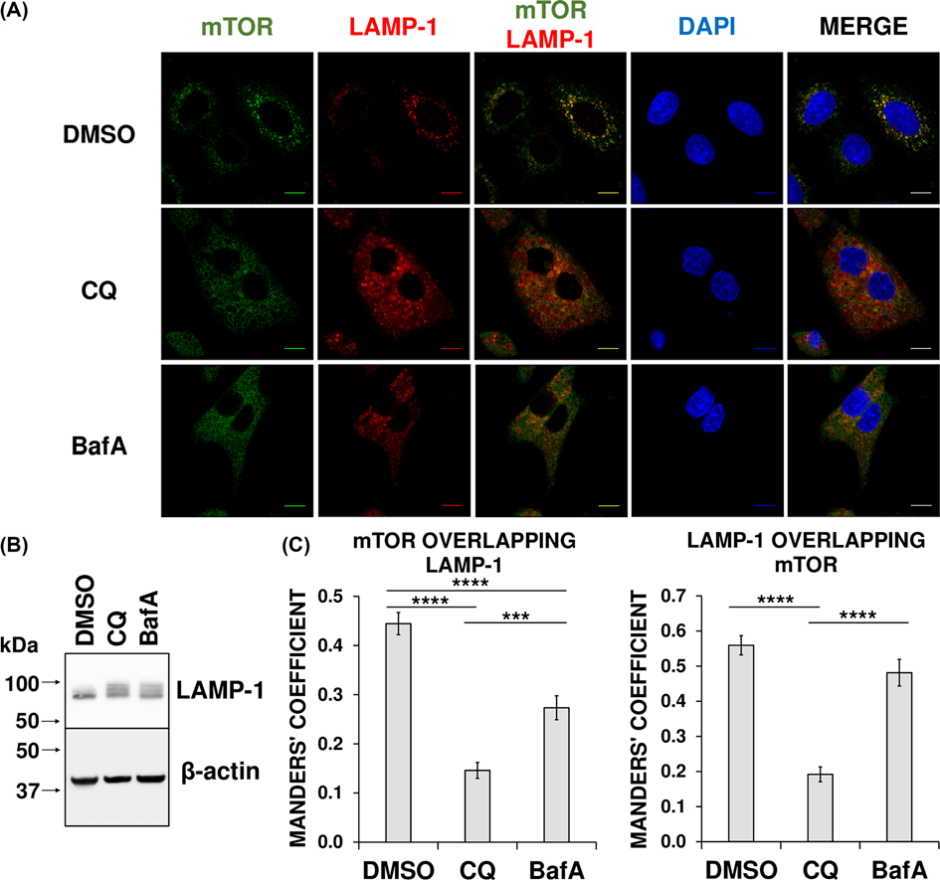
CQ and BafA treatment results in reduced localisation of mTOR with LAMP-1 in A549 cells (**A**) A549 cells were treated with 1:1000 DMSO (vehicle), 50 µM CQ, or 200 nM BafA for 23.5 h in Krebs–Ringer Bicarbonate buffer with 20 mM glucose, followed by a further 30-min treatment in DMSO, CQ, or BafA, but in DMEM + 10% FCS. All samples were then fixed and analysed via immunofluorescence. Each panel shows a set of cells labelled with DAPI (blue) and immunostained with antibodies to mTOR (green) and the lysosomal marker LAMP-1 (red). The scale bar represents 10 µm. (**B**) A549 cells were treated with 1:1000 DMSO, 50 µM CQ, or 200 nM BafA for 24 h. Cell extracts were prepared and then analysed via immunoblotting with the antibodies to LAMP-1 and β-actin. (**C**) Mander’s coefficients were determined and represented as the mean of at least ten cells per experimental sample. Results are representative of three independent experiments. Error bars represent ± standard error of the mean. Statistical significance was determined using unpaired Student’s t-test. Note: ***P≤0.001, ****P≤0.0001. Additional regions of interest are included in Supplementary Figure S3.

Confocal fluorescence microscopy revealed that CQ or BafA treatment each caused a similar increase in the area of sheep anti-LAMP-1 antibody staining as a result of larger lysosomal volume (Figure 4A). This is as expected, because each chemical inhibits lysosomal functions and induces the build-up of undigested macromolecules (or storage material) [28,29]. Indeed, an increase in LAMP-1 protein was confirmed via immunoblotting (Figure 4B). Under normal conditions, a proportion of mTOR co-localised with LAMP-1-stained lysosomes (Figure 4A). However, treatment with CQ or BafA ablated the punctate staining of mTOR and decreased the fraction of mTOR localised to the lysosome, with CQ causing a greater reduction than BafA (Figure 4A,C). Intriguingly, although the fraction of LAMP-1 localising with mTOR was also significantly reduced when cells were treated with CQ (demonstrating an overall reduction in co-localisation), this was not the case with BafA (Figure 4C). In more detail, staining patterns demonstrate a spatial exclusion of mTOR from LAMP-1 upon CQ treatment and this is confirmed with the respective Mander’s coefficients (Figure 4A,C). In contrast, with BafA treatment, although mTOR is no longer stained in a punctate manner as it is in the control (Figure 4A) and there is a statistically significant reduction in the proportion of mTOR associating with LAMP-1, the amount of LAMP-1 localising with mTOR did not change (Figure 4A,C). Unlike the situation for CQ, we cannot therefore conclude that BafA reduces mTOR/LAMP-1 co-localisation, when accounting for both Mander’s coefficients. However, these results might be confounded by the increase in total LAMP-1 (Figure 4B).

Nonetheless, whereas a fraction of mTOR localises with the lysosomal marker LAMP-1 under control conditions, such association is reduced when cells are treated with CQ or BafA. Thus, alkalinisation of the lumen of the lysosome leads to the dissociation of mTOR from the lysosomal surface, and, given the importance of lysosomal localisation for the activation of mTORC1 [7,8,11], this may account for the observed impairment of mTORC1 signalling.

### Increased amino acid levels fail to rescue the inhibition of mTORC1 signalling induced by lysosomal deacidification

mTORC1 activity is sensitive to amino acid availability, and to levels of amino acids within lysosomes [8], which may originate both from extracellular sources and lysosomal proteolysis. This raised the possibility that interfering with lysosomal function impaired the supply of intralysosomal amino acids to activate mTORC1. Therefore, we tested the possibility that increasing the supply of extracellular amino acids could restore mTORC1 activity in cells treated with CQ or BafA by compensating for a reduced supply of amino acids from intralysosomal proteolysis, this time by evaluating levels of p70S6K P-T389, which is the direct substrate of mTORC1 [30].

**Figure 5:**
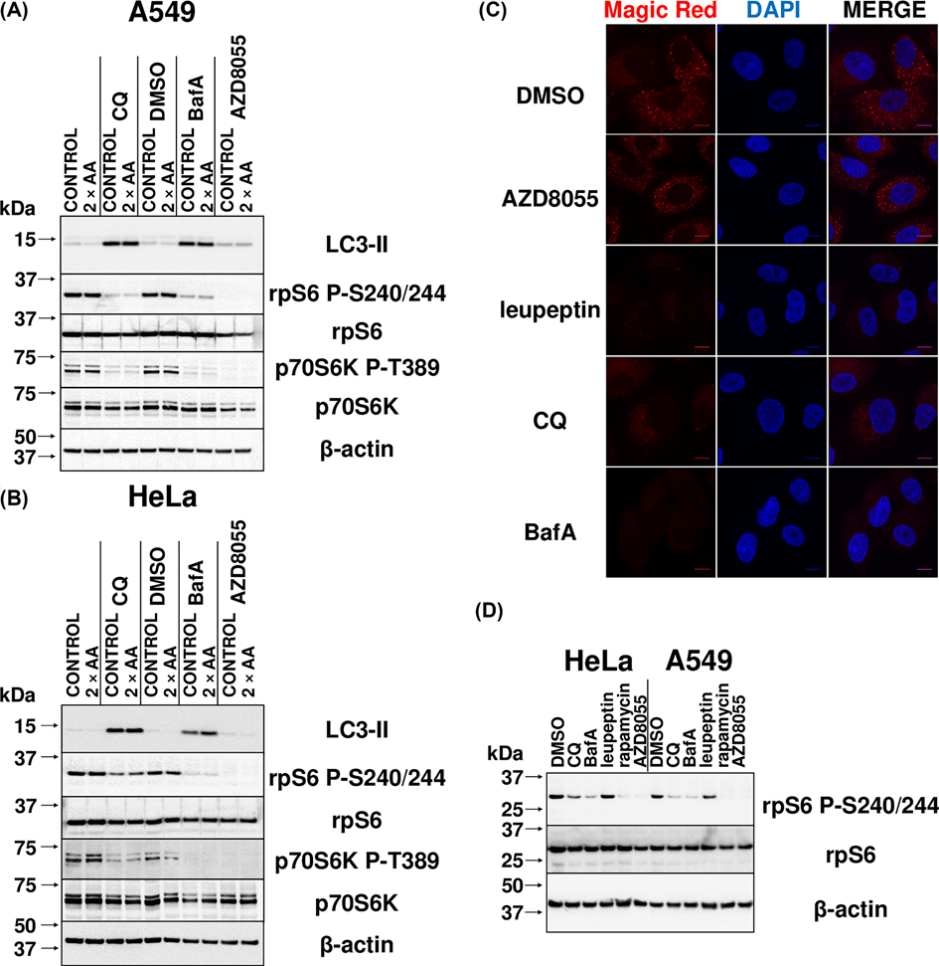
CQ or BafA-induced inhibition of mTORC1 signalling is not influenced by amino acid levels or intralysosomal proteolytic activity (**A**) A549 cells were left untreated or treated with 1:1000 DMSO (vehicle), 50 µM CQ, 200 nM BafA, or 1 µM AZD8055 for 23 h in DMEM + 10% FCS, followed by a further 1-h treatment in the same conditions with (2× AA) or without (CONTROL) the supplementation with 2× amino acids. Cell extracts were prepared and then analysed via immunoblotting with the indicated antibodies. (**B**) As with (A), but using HeLa cells. Results are representative of three independent experiments. (**C**) A549 cells were treated with 1:1000 DMSO, 50 µM CQ, 200 nM BafA, 50 µg/ml leupeptin, or 1 µM AZD8055 for 23 h. Culture medium was then supplemented with Magic Red for an additional hour. All samples were then fixed and analysed via confocal microscopy. Each panel represents a set of cells labelled with DAPI (blue) and Magic Red (red). The scale bar represents 10 µm. Additional regions of interest are displayed in Supplementary Figure S4. (**D**) A549 and HeLa cells treated with 1:1000 DMSO, 50 µM CQ, 200 nM BafA, 50 µg/ml leupeptin, 1 µM AZD8055, or 200 nM rapamycin for 24 h were analysed by immunoblotting with antibodies for total or phospho-rpS6 and β-actin. Results are representative of three independent experiments.

To examine the effects of increasing amino acid levels in the culture medium, A549 and HeLa cells were first treated with DMSO, CQ, BafA, or AZD8055 for 23 h. In one plate of cells in each treatment set, this was followed by supplementation of the medium for 1 h with essential and non-essential amino acids corresponding to those already present in DMEM to thus provide twice the concentration (Figure 5A,B). Immunoblotting of cell extracts revealed the expected increases in LC3-II levels upon lysosomal deacidification with CQ or BafA, regardless of the addition of amino acids. The addition of amino acids to cells that were already in complete medium did not significantly rescue the decreased phospho-S6 or phospho-p70S6K levels caused by CQ or BafA. It is therefore unlikely that lysosomal alkalinisation interferes with mTORC1 signalling by a mechanism related to amino acid supply.

### Inhibition of neither intralysosomal proteolytic activity alone nor autophagosome formation impairs mTORC1 signalling

We next assessed whether CQ and BafA decreased mTORC1 signalling due to their abilities to ablate intralysosomal proteolytic activity (which works optimally at low pH). To monitor such activity, we used the Magic Red cathepsin L system (manufactured by Bio-Rad). After cleavage by cathepsin L, this reagent is excited at 510–560 nm and emits at 570–620 nm, permitting an evaluation of intralysosomal protease cathepsin L activity in cells in culture, and providing a readout for overall intralysosomal activity.

A549 cells were first treated with the cysteine, serine, and threonine peptidase inhibitor leupeptin (a positive control for inhibition of lysosomal proteolysis [31]), the vehicle, CQ, BafA, or AZD8055 for 23 h, followed by supplementation of growth medium with Magic Red cathepsin L for a further hour. Cells were fixed and visualised by confocal fluorescence microscopy (Figure 5C). In control conditions, punctate staining consistent with the expected distribution of lysosomal cathepsin L activity was observed. A similar pattern was also observed in AZD8055-treated cells; also noteworthy was the additional enlargement of the lysosome, which was expected on account of AZD8055’s ability to directly inhibit mTORC1 activity and thus promote TFEB transactivation and lysosomal biogenesis. Conversely, there was a marked reduction in red fluorescence when cells were exposed to CQ, BafA, or leupeptin, confirming their ability to (indirectly) inhibit intralysosomal proteolysis (Figure 5C).

Lysates of similarly treated A549 and HeLa cells were then immunoblotted with antibodies for total and phospho-rpS6 and β-actin. (Figure 5D). In summary, unlike CQ and BafA, leupeptin did not decrease rpS6 phosphorylation, indicating it did not affect mTORC1 signalling. It is therefore likely that neither CQ nor BafA reduces mTORC1 activity by (indirectly) inhibiting intralysosomal proteolysis and the supply of amino acids, consistent with the conclusions from the amino acid-supplementation experiments.

**Figure 6:**
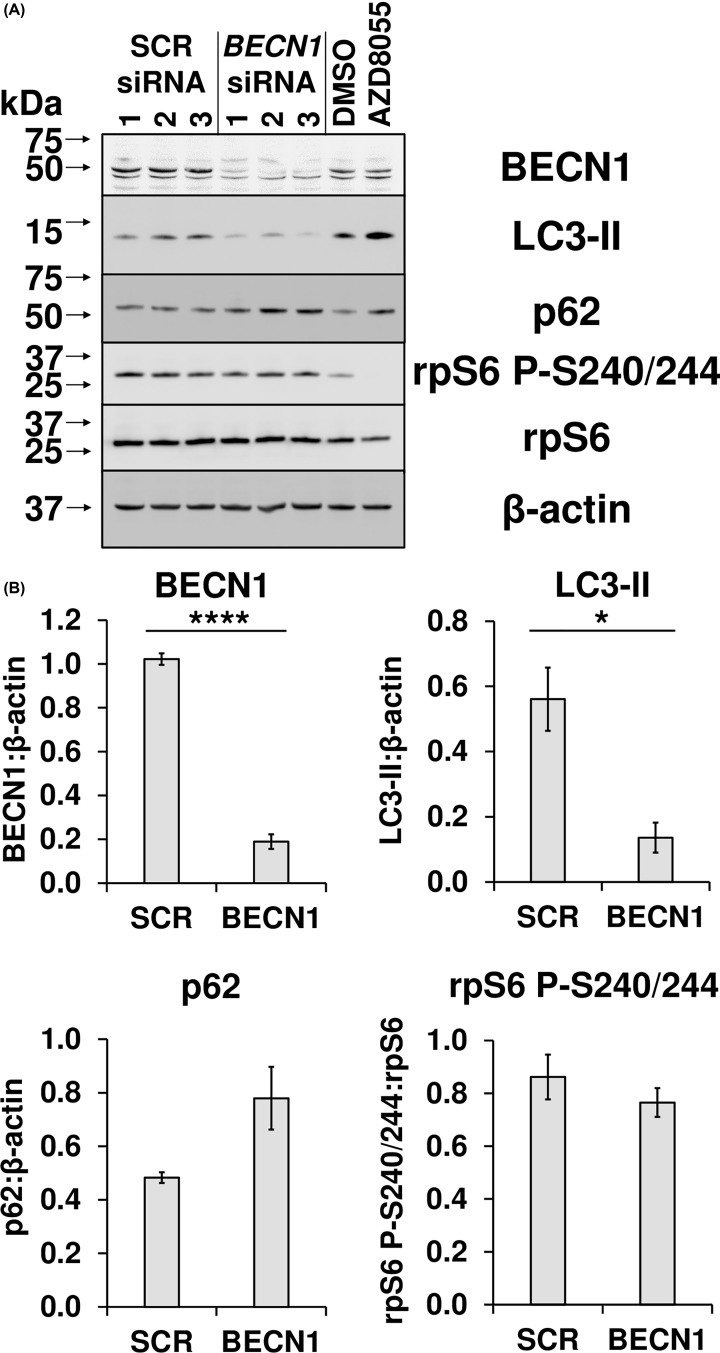
Inhibition of autophagosome formation alone does not interfere with mTORC1 signalling in A549 cells (**A**) Cell extracts from A549 cells transfected with the indicated concentration of either a scrambled siRNA (SCR) or one directed towards beclin-1 (*BECN1*); after 72 h, cells were lysed and samples were analysed via immunoblotting with antibodies to LC3-II, p62 (a protein that is targeted to and recycled in the autophagosome), total or phospho-rpS6 and β-actin. (**B**) BECN1, LC3-II, and p62 were quantified using densitometric analysis and normalised against β-actin, while the signal for rpS6 P-S240/244 was normalised against total rpS6, represented as the mean of the three biological replicates. Error bars represent ± standard error of the mean. Statistical significance was determined using unpaired Student’s t-test. Note: *P≤0.05, ****P≤0.0001.

Fusion of lysosomes with the autophagosome and degradation of the latter’s contents permits autophagic flux [31]. As mentioned previously, CQ and BafA inhibit this process even under basal conditions, resulting in accumulation of autophagosomes and material within them that would otherwise be degraded [31]. Another way of inhibiting autophagy involves reducing autophagosome number by depleting the expression of the *BECN1* mRNA and thus beclin-1 protein. A549 cells were transfected with scrambled siRNA or one directed to *BECN1* and, after 72 h, were analysed by immunoblotting with antibodies to LC3-II, p62 (a protein, encoded by *SQSTM1*, that is targeted to and degraded by the autophagosome), total and phospho-rpS6 and β-actin (Figure 6). The inhibition of autophagy was observed upon the knockdown of *BECN1* as shown by a statistically significant reduction in LC3-II levels and concomitant increase in p62 protein (*P=*0.0665). However, there was no change in phospho-rpS6 levels. Therefore, impairment of autophagy alone in conditions of nutrient abundance does not inhibit mTORC1 activity, and the effects of BafA and CQ on this pathway likely reflect another aspect of their effects on intralysosomal pH.

### Lysosomal storage of sucrose mimics lysosomal alkalinisation

Sucrose induces lysosomal stress in a range of primary and immortalised tissue culture lines, due to the combination of the rapid uptake of sucrose into the lysosome, and an inability to metabolise the sucrose on account of a lack of the enzyme invertase [32,33]. Therefore, not surprisingly, sucrose treatment results in cellular pathology typical of fibroblasts derived from LSD patients, including the increased nuclear localisation and activity of TFEB (an mTORC1 substrate), the consequent increased expression of the LAMP-1 protein, and up-regulated lysosomal biogenesis [4,33]. As such, sucrose-treated cells can serve as a model for LSDs, in which one or more metabolic enzymes are deficient, while other functions of the lysosome (such as protease activity) remain uninhibited.

Given that the ability of CQ and BafA to inhibit mTORC1 activity is not associated with their capacity to inhibit autophagy or an impairment of amino acid supply from lysosomal proteolysis, we wished to assess whether decreased lysosomal clearance and thus increased ‘storage’ of undegraded material within lysosomes might cause the impairment of mTORC1 signalling. We therefore evaluated whether sucrose mimicked the consequences of lysosomal alkalinisation. To this end, A549 and HeLa cells were treated with sucrose for 24, 48, or 72 h and then analysed by immunoblotting. In both lines, there was an increase in LAMP-1 protein when compared with untreated cells (Supplementary Figure S5A,B), indicating increased lysosomal biogenesis [4,33]. Concomitant increases in LC3-II and p62 are typically attributed to inhibition of autophagosome turnover [31], although these conclusions are complicated by the fact that *p62/SQSTM1* is also a TFEB target gene. Under normal conditions, mTORC1 is tethered to the cytoplasmic side of the lysosomal membrane and displays kinase activity towards Serine 142 and 211 in TFEB, which causes retention of TFEB in the cytoplasm [3]. Under conditions of cellular stress, such as nutrient starvation or lysosomal stress, mTORC1 is released from the lysosomal membrane and cannot phosphorylate TFEB, thus permitting translocation of TFEB to the nucleus and increased expression of target genes linked to autophagy and lysosomal function [4,11,34–38]. To evaluate the notion that TFEB is activated, RNA derived from identically treated cells was analysed for *p62* transcript levels by qPCR (Supplementary Figure 5C).

In both cell lines, after 24 h, *p62* mRNA was increased and remained at higher levels at 48 and 72 h when compared with the untreated controls. Finally, a statistically significant but transient decrease in rpS6 phosphorylation was observed after 24 h in A549 cells, indicating a reduction in mTORC1 activity, which had returned to control levels by 24 h (Supplementary Figure S5A,B). In HeLa cells, however, not only were levels of rpS6 P-S240/244 unchanged at 24 h, but actually increased by 48 and 72 h.

**Figure 7:**
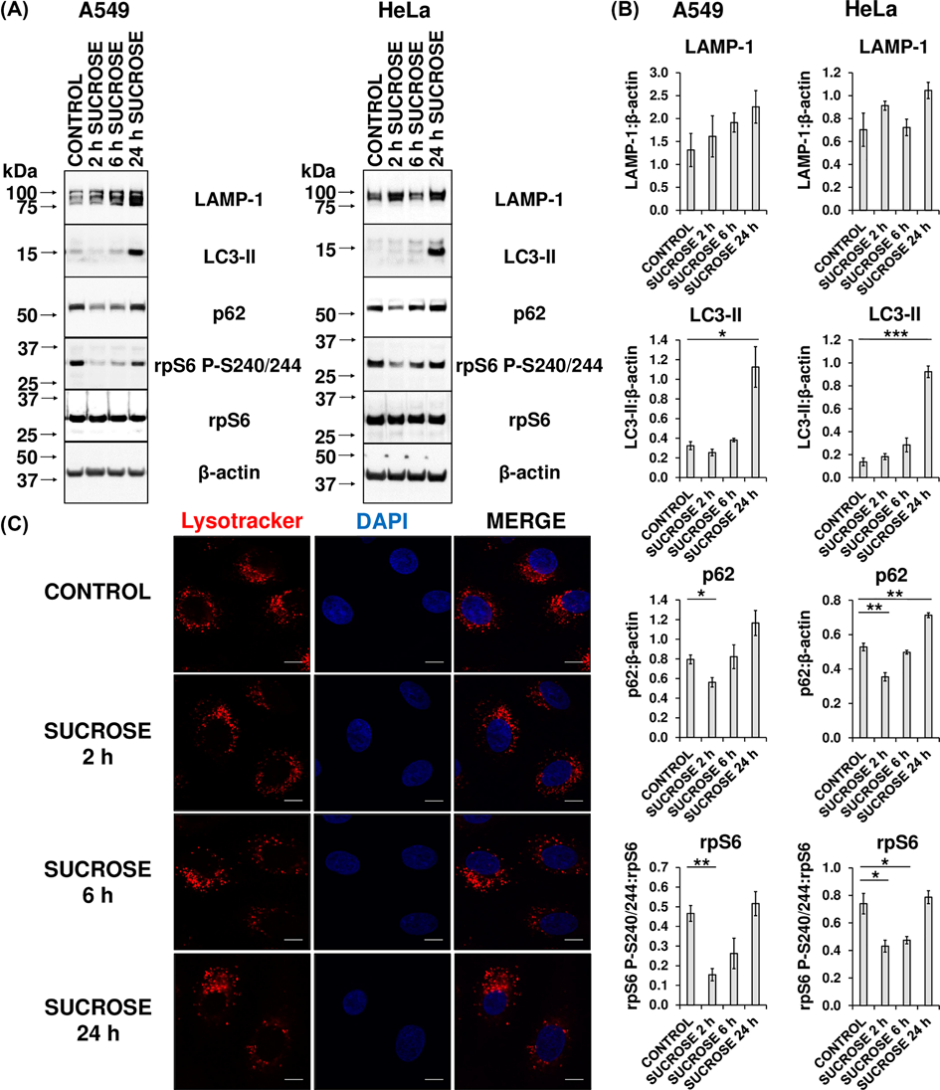
Sucrose treatment induces lysosomal dysfunction (**A**) Extracts were prepared from HeLa and A549 cells that had been treated with 100 mM sucrose for 2, 6, or 24 h or left untreated (CONTROL). Cell lysates were analysed by immunoblotting with the indicated antibodies. Results are representative of three independent experiments. (**B**) LAMP-1, LC3-II, and p62 were quantified using densitometric analysis and normalised against β-actin, while the signal for rpS6 P-S240/244 was normalised to total rpS6. Data are presented as the mean of the three biological replicates. Error bars represent ± standard error of the mean. Statistical significance was determined using unpaired Student’s t-test. Note: *P≤0.05, **P≤0.01. (**C**) A549 cells were treated with 100 mM sucrose for 2, 6, or 24 h or left untreated (CONTROL). Culture medium was additionally supplemented with LysoTracker Red DND-99 for 1 h prior to fixing. All samples were then analysed via confocal microscopy. Each panel represents a set of cells labelled with DAPI (blue) and LysoTracker Red DND-99 (red). The scale bar represents 10 µm.

We therefore speculated that whereas lysosomal storage (and consequent mTORC1 inactivation) induced by CQ or BafA would initially require the inhibition of metabolic enzymes and then a gradual accumulation of undegraded macromolecules, the effect of sucrose would be more rapid as the uncleared material (sucrose) is, in this instance, delivered directly to the lysosome. Indeed, this proposed scenario is consistent with the previously observed rapid translocation of TFEB from the cytoplasm to the nucleus in HeLa cells when similarly treated [4]. Furthermore, since it was possible that the effects of sucrose on mTORC1 signalling were also still transient, but more rapid, in HeLa cells, we treated both HeLa and A549 cells for shorter times (2, 6, as well as 24 h) with sucrose. This treatment (‘lysosomal storage’) resulted in the inactivation of mTORC1, as indicated by the significant reduction in phosphorylation of rpS6 P-S240/244 (when compared with total rpS6) after 2 or 6 h sucrose treatment in both cell lines (Figure 7A,B). Further supporting the notion that mTORC1 is inactivated at these time points, there was a decrease in p62 protein after 2 h of sucrose exposure (Figure 7A,B). It is likely that, at this acute stage, mTORC1 inactivity has derepressed ULK1-mediated inhibition of autophagy, but it is still too early for any concomitant TFEB-induced increase in p62 protein to be apparent. Nonetheless, provoking storage of material in lysosomes results in a rapid and transient inhibition of mTORC1 signalling in both cell types. Finally, to assess whether sucrose treatment caused alkalinisation of the lysosome, we employed LysoTracker Red DND-99. The extent of detection of LysoTracker-stained lysosomes was unaltered by sucrose treatments at all time points, ruling out that sucrose affects mTORC1 signalling by raising intralysosomal pH (Figure 7C).

In summary, the mTORC1 inactivation in response to BafA or CQ is likely due to the accumulation of uncleared material within the lysosome as an indirect consequence of intralysosomal alkalinisation (initially due to impairment of the activity of acidophilic lysosomal enzymes).

### CQ enhances levels of mRNAs for lysosomal and autophagy-related proteins only in A549 cells

**Figure 8:**
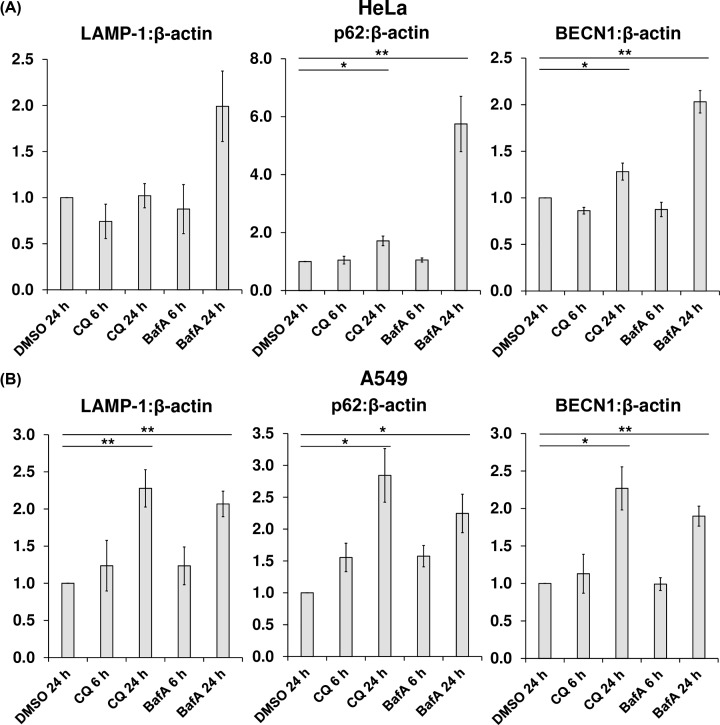
CQ enhances levels of mRNAs for lysosomal and autophagy-related proteins in A549 but not HeLa cells (**A**) Total RNA was extracted from HeLa cells treated with 1:1000 DMSO (vehicle), 50 µM CQ, or 200 nM BafA, for 6 or 24 h and used as a template for the preparation of cDNA, which was then analysed via qPCR employing primers designed to amplify fragments of the coding sequences of the lysosomal membrane protein LAMP-1, the autophagy-related proteins BECN1 and p62, and the internal control (β-actin). (**B**) As for (A), but with A549 cells. Results are representative of three independent experiments, each performed in technical triplicate and compared against the vehicle only control (DMSO 24 h). Error bars represent ± standard error of the mean. Statistical significance was determined using unpaired Student’s t-test. Note: *P≤0.05, **P≤0.01.

To further evaluate the effects of CQ and BafA on the function of TFEB, HeLa and A549 cells were treated with DMSO, CQ, or BafA for 6 or 24 h. mRNA was then extracted for qPCR analysis of to the mRNAs for the TFEB targets *LAMP-1, BECN1*, and *p62*. After 24 h, in HeLa cells, BafA treatment resulted in a striking increase in the mRNA levels of all three direct TFEB targets (as a ratio against β-actin expression) when compared with those of the untreated control, which was also statistically significant for *p62* and *BECN1* (for LAMP-1, *P*=0.0604) (Figure 8A). However, although 24-h CQ treatment significantly increased *p62* and *BECN1* mRNA levels, it did so more modestly than BafA. In contrast, in A549 cells, both CQ and BafA treatment caused a similar and significant increase in TFEB target gene expression (Figure 8B). Thus, agents that cause intralysosomal alkalinisation, particularly BafA, activate transcription of TFEB target genes, consistent with the impairment of mTORC1 signalling.

### BafA enhances AMPK activity

In some cell types, a structural and mechanistic link has been established between AMPK and the v-ATPase [39,40]. AMPK is activated under conditions of reduced cellular ‘energy’ (ATP) levels, when AMP concentrations rise. AMPK can inhibit mTORC1 both directly (via phosphorylation of Serines 722 and 792 of RAPTOR, a key component of mTORC1) [41] and indirectly (through phosphorylation of Thr^1227^ and Ser^1345^ of TSC2, which in turn deactivates the mTORC1 activator, Rheb) [42]. AMPK also associates with and phosphorylates ULK1 at S317 and S777, thereby activating it [43–46]. As the function of the v-ATPase is inhibited by BafA, but not CQ, we deemed it relevant to evaluate whether these compounds differed in their ability to promote activation of AMPK, particularly in light of their differing effects on the expression of TFEB target genes in HeLa cells (Figure 8A).

First, to assess the possible role of AMPK in CQ- and/or BafA-induced mTORC1 inhibition, HeLa and A549 cells were treated with CQ, BafA, or AZD8055, or starved for amino acids for 30 min (after growth in serum-free medium for 23.5 h). Furthermore, in both cell lines, BafA caused a significant increase in the phosphorylation of S79 in ACC, an AMPK substrate, indicating activation of AMPK. However, CQ did not elicit this effect in HeLa cells, while in A549 cells, the CQ-induced increase in phospho-ACC was smaller than that caused by BafA. Intriguingly, at shorter durations of treatment (1, 2, or 6 h), neither CQ nor BafA affected ACC phosphorylation (Supplementary Figure S2). This indicates CQ and BafA have different effects on AMPK activity after 24 h. Given that both compounds impair mTORC1 signalling, mechanisms other than activation of AMPK are likely involved, especially for CQ.

## Discussion

Our data show CQ and BafA each cause impaired mTORC1 signalling in cells grown in abundant nutrient conditions, which includes extracellular amino acids. However, while de-acidification of the lysosomal lumen occurs rapidly (Figure 2), only longer term treatment of human cells with CQ or BafA results in impairment of mTORC1 activity (Figure 1 and Supplementary Figure S2), suggesting such inhibition is a consequence of alkalinisation rather than altered pH being the proximal cause. This is likely at least in part a consequence of the decrease in localisation of mTOR with LAMP1-stained lysosomes, given that the lysosomal surface is a major locus for the activation of mTORC1 [8] (Figure 4). Additionally, although CQ and BafA each inhibit autophagy by increasing intralysosomal pH, it is unlikely that inhibition of autophagy is itself responsible, as siRNA-mediated knockdown of *BECN1* did not affect mTORC1 signalling (Figure 6). Furthermore, as leupeptin (which inhibits cysteine, serine and threonine peptidases without altering lysosomal pH [31]) did not alter phospho-rpS6 levels (Figure 5C,D), it is also unlikely that ablated intralysosomal proteolysis and any associated depletion of intralysosomally generated amino acids impair mTORC1 signalling. This conclusion is supported by our observation that increased supply of extracellular amino acids failed to rescue the inhibition of mTORC1 signalling caused by either CQ or BafA (Figure 5A,B).

Rather, experiments utilising sucrose treatment (an established model for LSDs) [32,33] suggest that the accumulation of undegraded substrates within lysosomes is the link between the intralysosomal alkalinisation caused by CQ and BafA and inactivation of mTORC1 (Figure 7 and Supplementary Figure S5). Two observations from these data nonetheless require qualification. Firstly, CQ- and BafA-induced inhibition of mTORC1 requires a longer duration of treatment than does exposure to sucrose. This may reflect an indirect effect of CQ and BafA whereby they induce lysosomal storage (and consequent mTORC1 inactivation) secondary to lysosomal alkalinisation and inhibit metabolic enzymes causing the gradual accumulation of undegraded macromolecules, whereas sucrose-induced ‘storage’ is quicker as the storage material is sucrose itself. This notion is supported by the observation in in earlier studies [4] that sucrose induces the rapid translocation of TFEB from the cytoplasm to the nucleus. Secondly, at longer time points, there is an increase in p62 and LC3-II proteins (Supplementary Figure S5A,B), together suggesting an inhibition of autophagy, as well as an increase in the mRNA levels of *p62* (Supplementary Figure S5C), which are typically associated with *enhanced* TFEB activity and autophagy. Two contrasting processes thus appear to be occurring: an inhibition of autophagic flux and a transcriptional response associated with increased expression of lysosomal and autophagy genes. Previously, in CA1 pyramidal neurons of the hippocampus from Alzheimer’s disease patients, an up-regulation of the network of genes regulated by TFEB was observed, along with a decrease in autophagic flux due to an inability to clear the accumulated substrate [47]. Therefore, our data with respect to sucrose treatment of A549 and HeLa cells provide a further similarity between lysosomal alkalinisation and substrate storage caused by reduced lysosomal clearance.

In summary, our results using sucrose are consistent with previous observations of a TFEB-induced (and thus mTORC1-regulated) transcriptional response to lysosomal dysfunction [4,11]. This is conceptually novel as we propose that CQ and BafA ultimately exert their effects on the endo-lysosomal network in a similar mechanistic fashion to that of accumulation of substrates within the lysosome. Indeed, altered mTORC1 signalling has commonly been demonstrated as a component of diseases associated with lysosomal dysfunction. These include (with the mutated gene in parentheses): juvenile neuronal ceroid lipofuscinosis (*CLN3*) [48]; Scheie syndrome (*IDUA*) [49]; aspartylglucosaminuria (*AGA*) [49]; Fabry disease (*GLA*) [49,50]; cystinosis (*CTNS*) [51]. In the final case, the scenario is complicated by the authors’ finding that the product of *CTNS* interacts with the v-ATPase–Ragulator–RagGTPase complex, so the effect in this example is more likely to be a direct one. In another recent study, mTORC1 signalling is impaired in myotubes into which a mutation linked to Pompe’s disease (a genetic disorder in which lysosomal function is affected by deficiency in the enzyme α-glucosidase, which normally breaks down glycogen within lysosomes) has been introduced [52]. However, this is a quite distinct situation as mTOR remains associated with lysosomes (on account of the hyperactivation of the v-ATPase) but instead its negative regulator, TSC2, is recruited to these organelles to inactivate mTORC1. Overall, therefore, rather than being described merely as inhibitors of autophagy, it would be more appropriate to consider CQ and BafA, like sucrose, as mimetics of LSDs at the cellular level, as, in addition, they also promote certain aspects of autophagic flux (such as TFEB activation) via mTORC1 inactivation.

**Figure 9:**
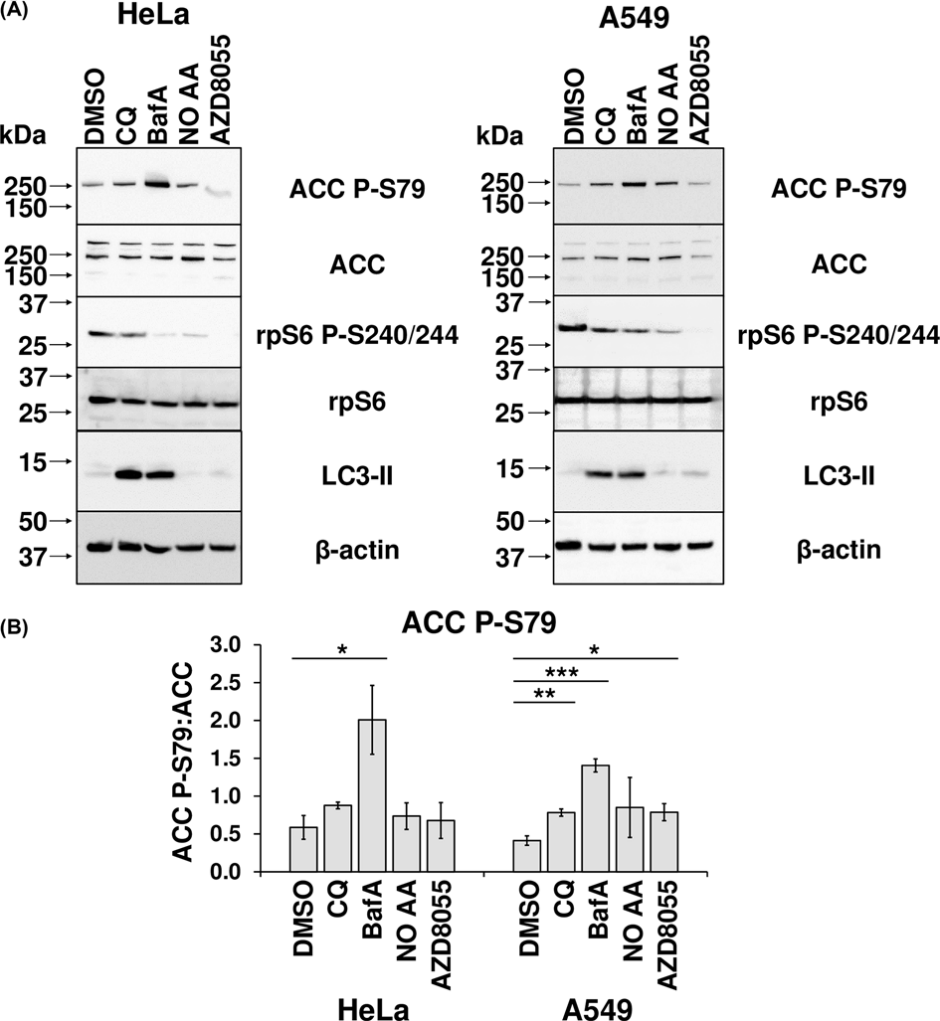
BafA enhances AMPK activity (**A**) Extracts were prepared from HeLa and A549 cells that had been treated with 1:1000 DMSO (vehicle), 50 µM CQ, 200 nM BafA, or 1 µM AZD8055 for 24 h. Alternatively, they were treated with serum-free DMEM for 24 h or for 23.5 h, followed by a 30-min treatment with Krebs–Ringer Bicarbonate buffer 20 mM glucose, but no added amino acids (NO AA). Cell lysates were analysed via immunoblotting with the indicated antibodies. Results are representative of three independent experiments. (**B**) ACC P-79 (relative to total ACC) was quantified by densitometric analysis, and represented as the mean of three independent experiments. Error bars represent ± standard error of the mean of the indicated number of independent experiments performed. Statistical significance was determined using unpaired Student’s t-test. Note: *P≤0.05, **P≤0.01, ***P≤0.001.

Additional considerations are raised by our observations of greater inhibition of mTORC1 activity by BafA than CQ, with respect to reduced rpS6 phosphorylation (Figures 1B, 5B,D, and 9A) and increased mRNA levels of TFEB target genes (Figure 8A) in HeLa cells. The fact that CQ impairs mTORC1 signalling indicates that lysosomal alkalinisation itself impacts on mTORC1 signalling, while the somewhat greater effect of BafA might indicate that interference with the v-ATPase, a partner for Ragulator, exerts additional effects on mTORC1 [8]. Furthermore, while 24-h BafA treatment also caused a significant increase in AMPK activity (seen as phosphorylation of ACC S79) in both cell lines (Figure 9), a similar effect for CQ (albeit modest, when compared with that elicited by BafA) was only evident in A549 cells (Figure 9). In HeLa cells it appears that inhibition of the v-ATPase itself, rather than lysosomal alkalinisation, is a trigger for AMPK activation in this line. As BafA quickly promotes lysosomal de-acidification (Figure 2), it was surprising that shorter durations of treatment with BafA (1–6 h) failed to alter ACC phosphorylation and thus AMPK activation (Supplementary Figure S2), because a more rapid response to BafA would be consistent with recent observations that link the v-ATPase to activation of AMPK [39]. During nutrient stress, a complex consisting of AXIN and LKB1 interacts with the v-ATPase, promoting the phosphorylation of AMPK by LKB1 and thus the activation of AMPK [39]. However, as A549 and HeLa cells do not express detectable levels of LKB1 [53,54], this mechanism cannot operate in them, perhaps explaining why longer BafA treatment is required to activate AMPK. The reason that BafA activates AMPK, while CQ only does so to a much more limited extent, probably reflects the fact the former affects the v-ATPase directly, while CQ does not [39]. At any rate, independent of the exact mechanism, the chronic effect of BafA to inhibit mTORC1 may ultimately involve both activation of AMPK and lysosomal alkalinisation, while CQ, by exerting only the latter effect, impairs mTORC1 signalling more modestly. A similar pattern was seen for the abilities of CQ and BafA to up-regulate known TFEB targets; in HeLa cells, BafA treatment for 24 h led to increased levels of three known direct TFEB targets (LAMP-1, BECN, and p62), whereas CQ caused only a modest up-regulation of p62 and BECN1 (Figure 8A). There may also be differences between cell lines (Figures 8B and 9). In summary, lysosomal alkalinisation is sufficient to inhibit mTORC1 activity, although additional signalling mechanisms likely contribute to the stronger responses to BafA. Indeed, it should be noted that other differences have been seen between the effects of CQ and BafA, such as on the organisation of certain organelles, e.g., the Golgi [13]. This factor may give rise to differences in their overall effects. However, the common means by which both compounds act as mimetics of acute LSDs is via their shared capacity to induce the alkalinisation of the lumen of the lysosome, the accumulation of intralysosomal storage material (the defining characteristic of LSDs) and subsequently also the impairment of mTORC1 activity.

Although, an inhibitory effect of lysosomal alkalinisation on mTORC1 signalling has been previously reported [55], those authors proposed that impeding autophagy itself inhibits mTORC1 in an attempt to compensate by promoting autophagy, in a type of ‘homoeostatic’ feedback mechanism; this scenario is not supported by our data, as the inhibition of autophagy by siRNA-mediated knockdown of *BECN1* did not influence mTORC1 signalling (Figure 6). We note that there is also a contrasting report that, in osteoclasts, alkalinisation of the lysosome (via exposure to CQ or ammonium chloride) *increases* mTORC1 activity, an effect which was associated with increased mTOR protein levels [56]. Enhanced mTORC1 activity resulting from increased lysosomal pH caused by treatment with CQ and (more potently) BafA has also been observed in chondrocytes (but without an increased level of mTOR protein) [57]. These findings may reflect a specific feature of cells that are related to bone growth.

In conclusion, future studies using BafA or CQ (for example, to manipulate lysosomal function/autophagy) must take into account their abilities to modulate mTORC1 signalling and to acutely mimic LSDs (and in the case of BafA, to further activate AMPK), since these pathways exert diverse effects on cell physiology [1]. This is becoming more relevant medically, given that recent research implicates impaired v-ATPase activity (due to genetic defects) and increased intralysosomal pH in more highly prevalent conditions such as late-onset neurodegenerative disorders [58].

## Supplementary Material

Supplementary Figures S1-S5

## Abbreviations

ACC: acetyl-CoA carboxylase
AMPK: 5′-adenosine monophosphate-activated protein kinase
BafA: bafilomycin A1
BECN1: beclin-1
BSA: bovine serum albumin
cDNA: complementary DNA
CQ: chloroquine
CTNS: cystinosis
DMEM: Dulbecco’s modified Eagle medium
LAMP-1: lysosomal-associated membrane protein 1
LC3: microtubule-associated proteins 1A/1B light chain 3B
LKB1: liver kinase B1
LSD: lysosomal storage disorder
MEM: minimal essential medium
mTORC1: mammalian target of rapamycin complex 1
p70S6K: ribosomal protein S6 kinase
PFA: paraformaldehyde
qPCR: quantitative real-time PCR
Rheb: ras homologue enriched in brain
rpS6: ribosomal protein S6
SQSTM1: sequestosome-1
TFEB: transcription factor EB
TSC: tuberous sclerosis
ULK1: Unc-51 like autophagy activating kinase 1
v-ATPase: vacuolar type H^+^-ATPase
