# Supplementary material for: Chloroquine and bafilomycin A mimic lysosomal storage disorders and impair mTORC1 signalling
Source: Biosci Rep. 2020 Apr 28;40(4):BSR20200905. doi: 10.1042/BSR20200905 (PMC7189491; doi:10.1042/BSR20200905)
Supplement: Supplementary Figures S1-S5 [file BSR-2020-0905_supp.pdf]

Supplementary Figure 1.

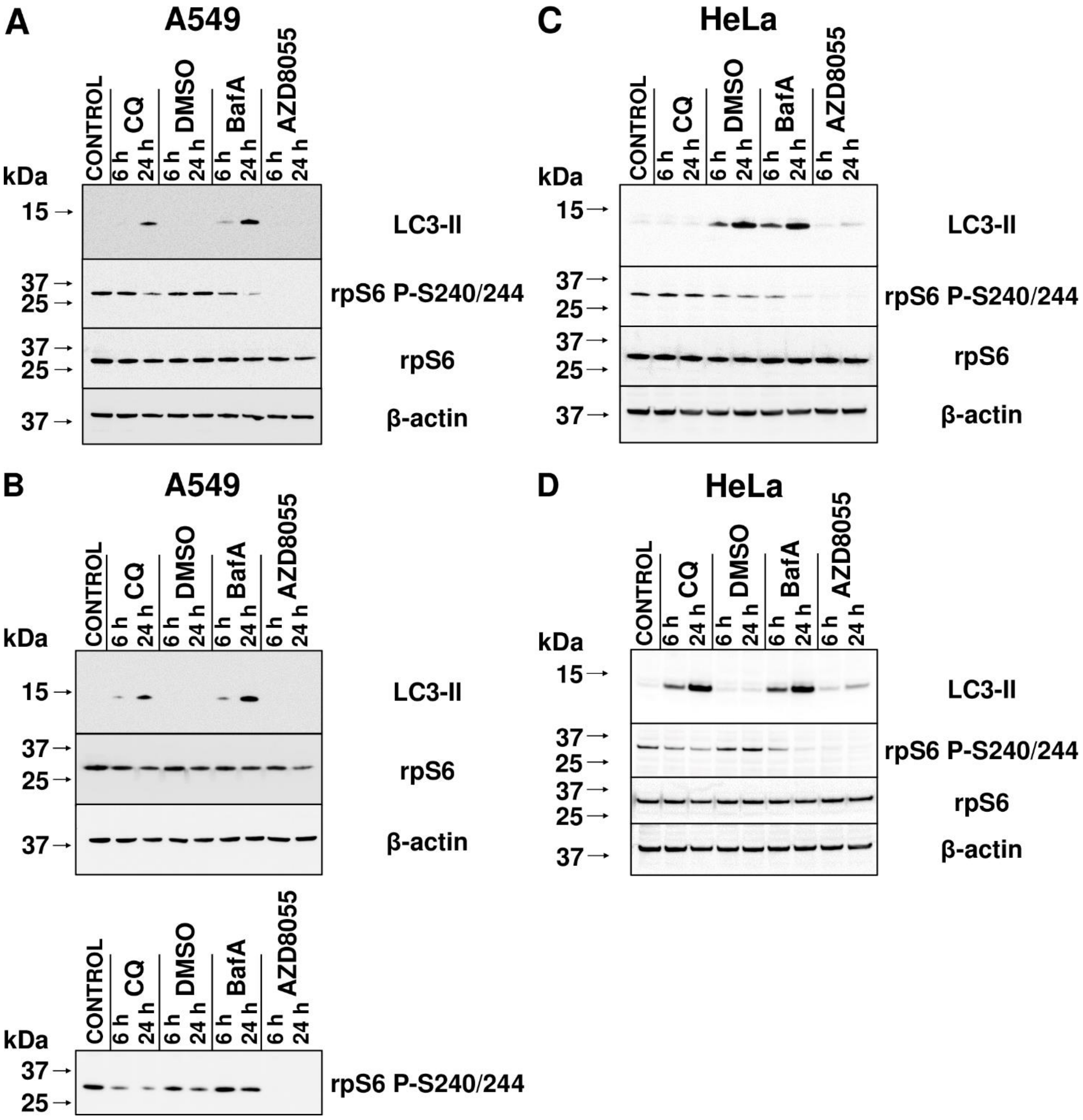

Supplementary Figure 1. Replicate experiments from Figure 1. **A.** and **B.** Replicate experiments in A549 cells. **C.** and **D.** Replicate experiments in HeLa cells. See legend to Figure 1 for further information. Please note alternative loading order when compared with Figure 1 in **B** (rpS6 P-240/244 immunoblot only) and **C**.

Supplementary Figure 2.

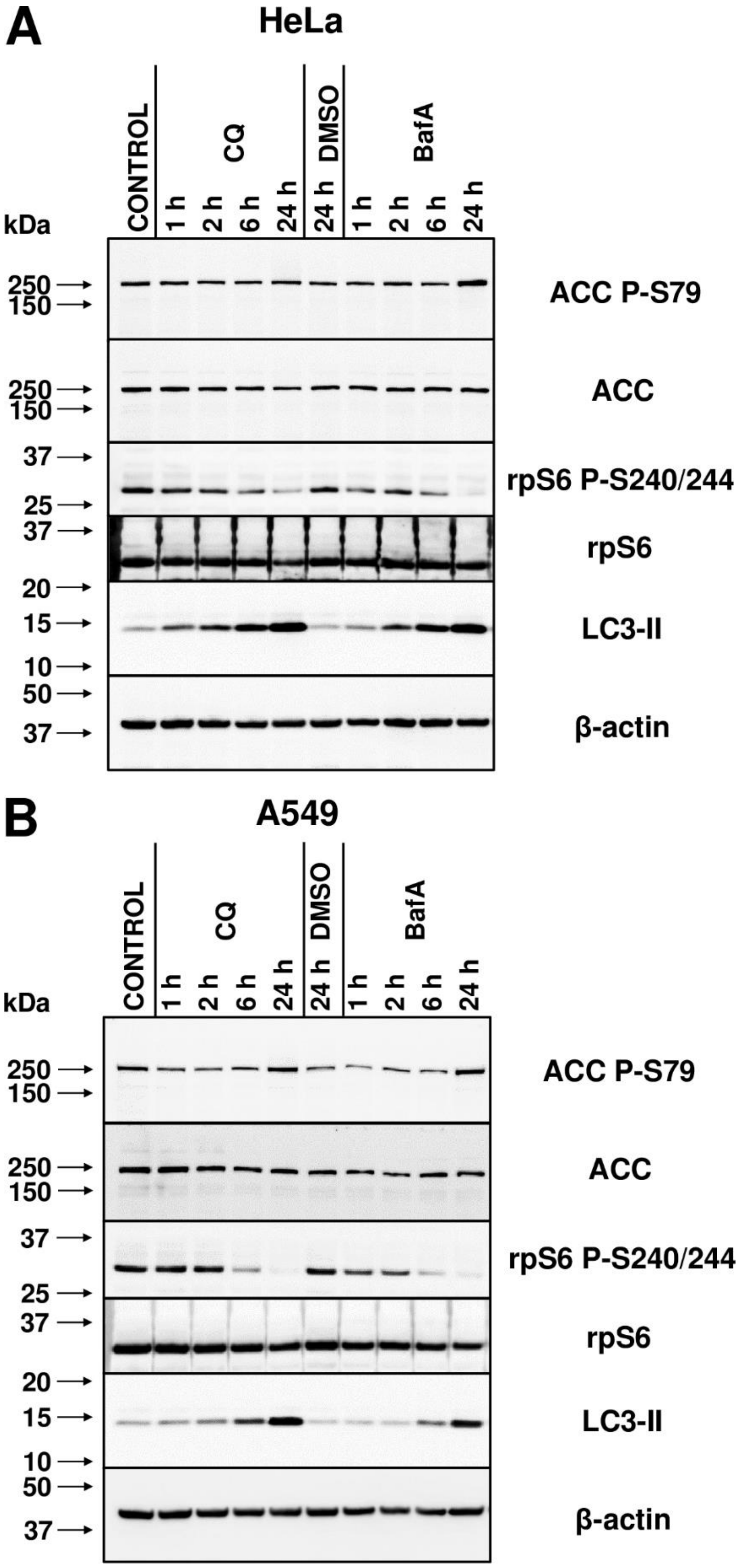

Supplementary Figure 2. Acute treatment with neither CQ nor BafA enhances AMPK signalling. A. Extracts were prepared from HeLa and A549 cells that had been treated with either 50 µM CQ or 200 nM BafA for 1, 2, 6 or 24 h. Alternatively, they were treated with 1:1000 DMSO for 24 h or left untreated (CONTROL). Cell lysates were analysed via immunoblotting with the indicated antibodies. **B.** As for **A**, but with A549 cells. Results are representative of three independent experiments.

Supplementary Figure 3.

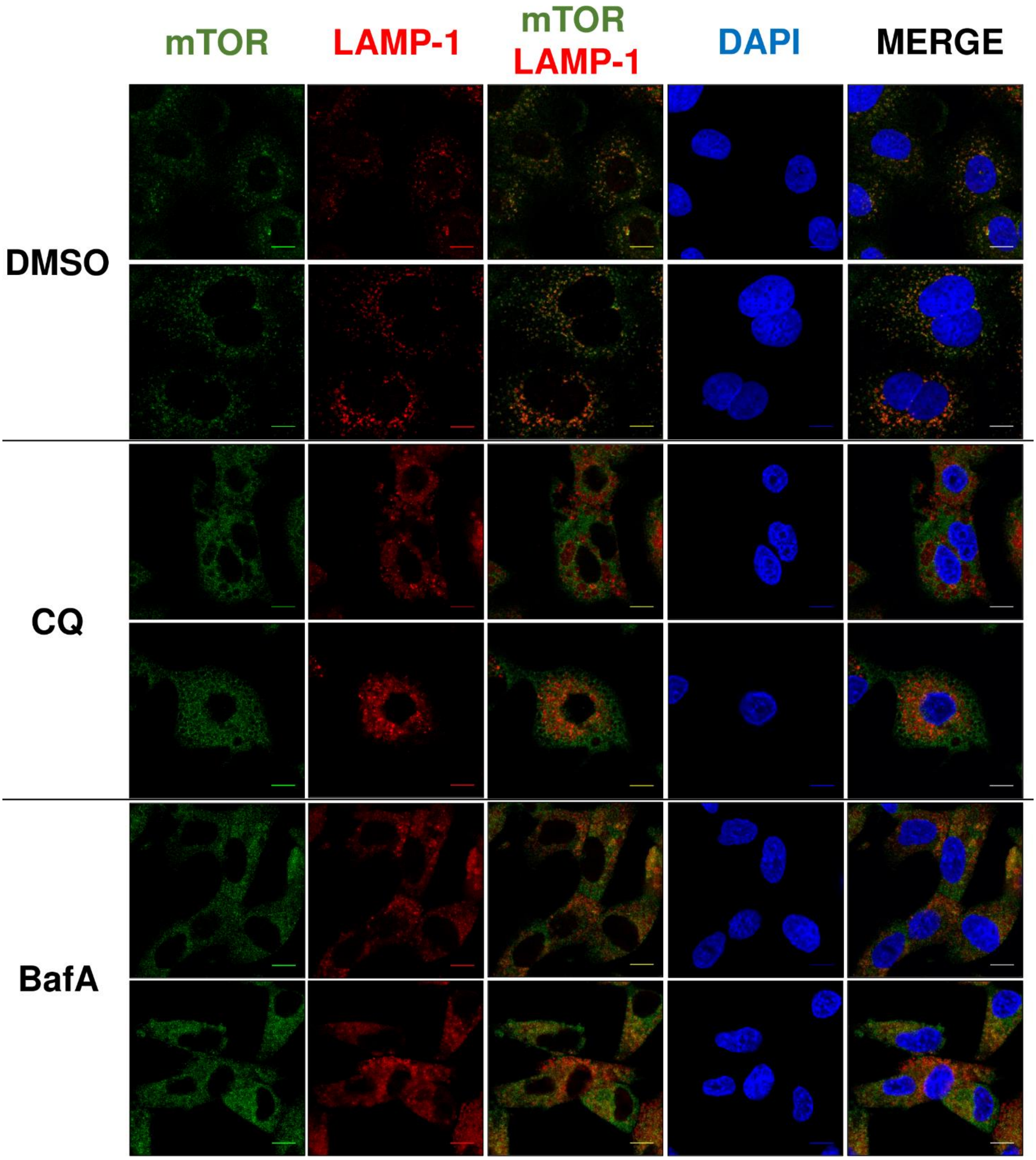

Supplementary Figure 3. Additional regions of interest from Figure 4A. See legend to Figure 4A for further information.

Supplementary Figure 4.

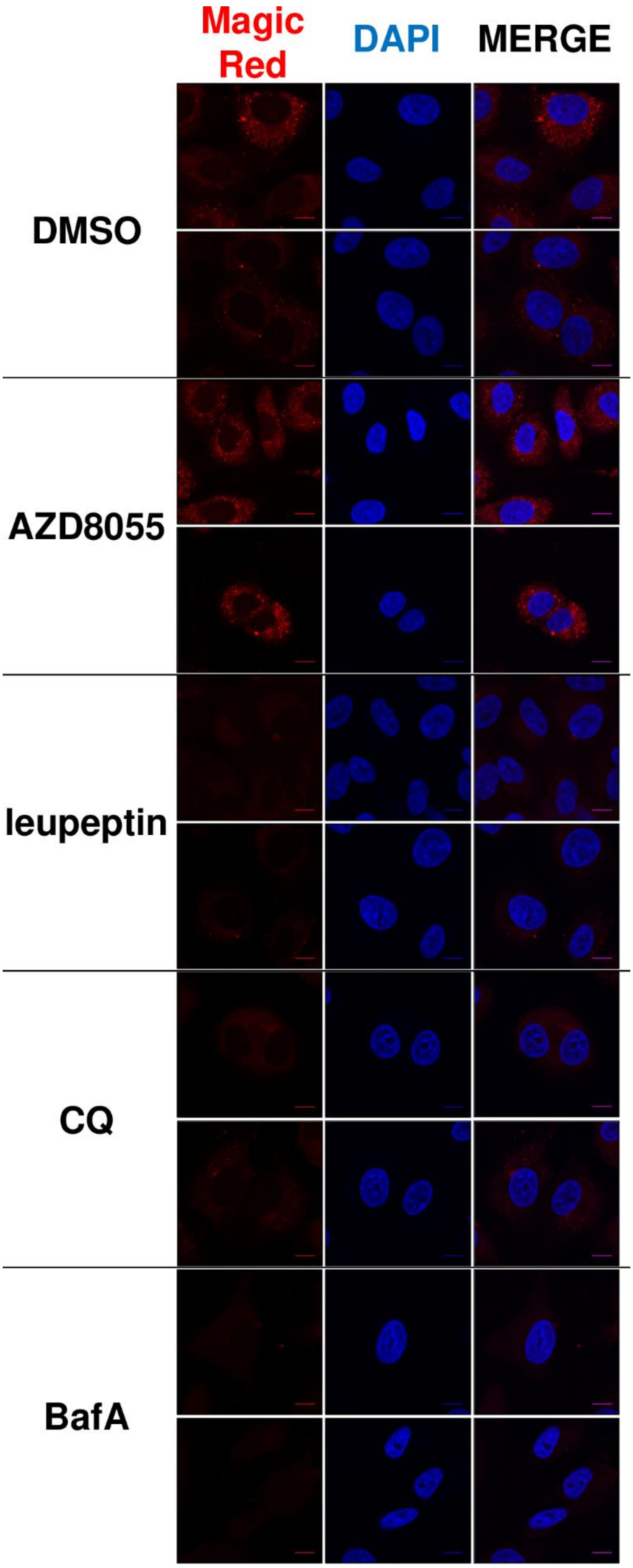

Supplementary Figure 4. Additional regions of interest from Figure 5C. See legend to Figure 5C for further information.

Supplementary Figure 5.

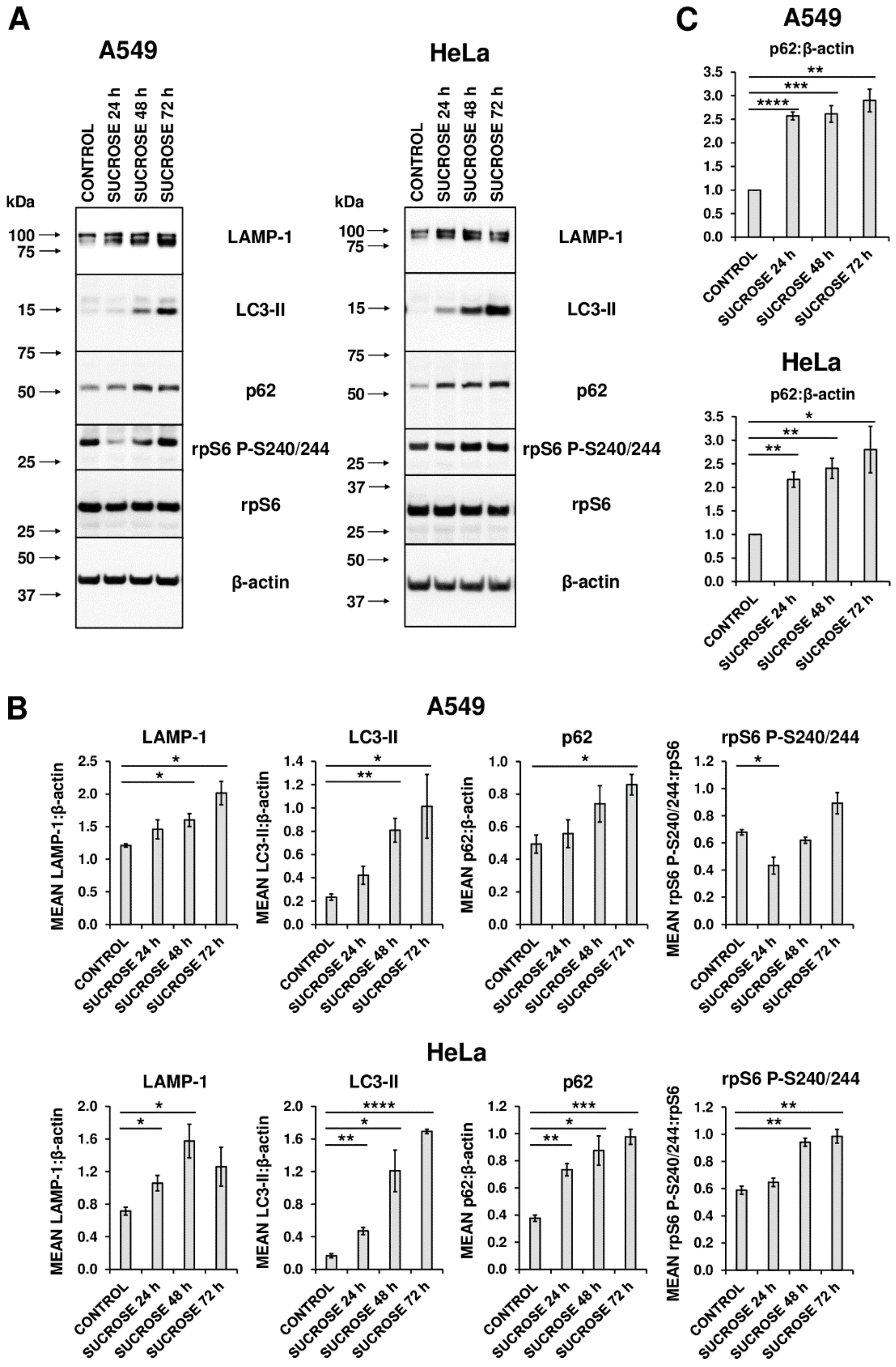

Supplementary Figure 5. Sucrose treatment mimics lysosomal alkalinisation. A. Extracts were prepared from HeLa and A549 cells that had been treated with 100 mM sucrose for 24, 48 or 72 h or left untreated (CONTROL). Cell lysates were analysed by immunoblotting with the indicated antibodies. Results are representative of three independent experiments**. B.** LAMP-1, LC3-II and p62 were quantified using densitometric analysis and normalised against β-actin, while the signal for rpS6 P-S240/244 was normalised to total rpS6, represented as the mean of the three biological replicates. C. Total RNA extracted from cells treated as in **A** was used as a template for the preparation of complementary DNA, which was then analysed via quantitative real-time PCR employing primers designed to amplify fragments of the coding sequences of *p62*, and the internal control (β-actin). Results are representative of three independent experiments, each performed in technical triplicate and compared against the untreated control.
